# Physiological Resonance in Empathic Stress: Insights from Nonlinear Dynamics of Heart Rate Variability

**DOI:** 10.3390/ijerph18042081

**Published:** 2021-02-21

**Authors:** Estelle Blons, Laurent M. Arsac, Eric Grivel, Veronique Lespinet-Najib, Veronique Deschodt-Arsac

**Affiliations:** 1 Univ. Bordeaux, CNRS, Laboratoire IMS, UMR 5218 Talence, France; laurent.arsac@u-bordeaux.fr (L.M.A.); veronique.arsac@u-bordeaux.fr (V.D.-A.); 2 Bordeaux INP, Univ. Bordeaux, CNRS, Laboratoire IMS, UMR 5218 Talence, France; Eric.Grivel@enseirb-matmeca.fr (E.G.); veronique.lespinet@ensc.fr (V.L.-N.)

**Keywords:** empathic stress, resonance, heart rate variability, complexity, multiscale entropy

## Abstract

Because most humans live and work in populated environments, researchers recently took into account that people may not only experience first-hand stress, but also second-hand stress related to the ability to empathically share another person’s stress response. Recently, researchers have begun to more closely examine the existence of such empathic stress and highlighted the human propensity to physiologically resonate with the stress responses of others. As in case of first-hand stress, empathic stress could be deleterious for health if people experience exacerbated activation of hypothalamic–pituitary–adrenal and autonomic nervous systems. Thus, exploring empathic stress in an observer watching someone else experiencing stress is critical to gain a better understanding of physiological resonance and conduct strategies for health prevention. In the current study, we investigated the influence of empathic stress responses on heart rate variability (HRV) with a specific focus on nonlinear dynamics. Classic and nonlinear markers of HRV time series were computed in both targets and observers during a modified Trier social stress test (TSST). We capitalized on multiscale entropy, a reliable marker of complexity for depicting neurovisceral interactions (brain-to-heart and heart-to-brain) and their role in physiological resonance. State anxiety and affect were evaluated as well. While classic markers of HRV were not impacted by empathic stress, we showed that the complexity marker reflected the existence of empathic stress in observers. More specifically, a linear model highlighted a physiological resonance phenomenon. We conclude on the relevance of entropy in HRV dynamics, as a marker of complexity in neurovisceral interactions reflecting physiological resonance in empathic stress.

## 1. Introduction

In humans, a number of daily-life activities act as stressors to which people react in an individual manner to provide the organism with sufficient motivation and energy to cope with adverse constraints. Although everyday stressors rarely represent clear traumatic events, the persistent exposure to mild stress durably challenges a number of organic functions, which may lead to permanent psychophysiological dysregulations. So far, individual experience of stress and anxiety have been explored in a broad spectrum of research among psychologists, physiologists and clinicians, but surprisingly, a collective experience of stress has been addressed only for the last years.

Since most human beings live in a social environment, stress experiences are not restricted to the challenges faced by each one individually. Since 2012, attention has been paid to second-hand stress in individuals exposed to the stress of another, bringing out the emerging area of empathic stress and taking up the topic of peripheral-physiological resonance in the context of stress research [1,2,3,4]. It has been argued that human capacity for empathy enables an observer to resonate with the responses of a target under stress, resulting in a physiological linkage between the two. Empathy is a multifaceted process recognized to involve the sharing of an individual’s affective and physiological states and the conscious understanding of what the other individual knows, thinks or feels [2,5,6,7,8,9,10,11], as evidenced by concomitant neural processing mapped by neuroimaging [5,9,12,13,14,15,16]. The role of neurophysiological backgrounds of empathy has fostered the concept of physiological resonance [1,2].

Experiments involving a mother and her child or romantic partners showed that social proximity might exacerbate empathic stress and physiological resonance [17,18,19]. Recent works show that mother’s stress level assessed through activation of sympathetic autonomous responses triggered a rise in the heart rate of their children [20,21]. Autonomic functions in physiological resonance become less clear when both parasympathetic and sympathetic responses are evaluated concomitantly [21]. Apart the use of electrodermal activity (EDA) as a pure sympathetic marker in marital couples, other indices assess physiological resonance between targets and observers [1,2,22]. Mostly, examinations of the hypothalamic–pituitary–adrenal axis activity made it possible to confirm endocrine stress contagion through resonance of cortisol activity in dyadic relationships [3,18,19]. Endocrine-based markers of a physiological response to stress help draw a delayed picture of resonance. In contrast, nervous autonomic markers react more closely to the task at hand. This provides a different hallmark focused on concurrent physiological resonance, which is not blind to short-term fluctuations. Overall, because of a quite limited number of studies having scrutinized physiological resonance and the diversity of employed methodologies, the underlying mechanisms of physiological resonance remain unclear to date. Rather, in most research, empathy has served as a paradigm to explore the conditions in which second-hand stress arises in a person during social interactions [1,2], which brought about no clear physiological background for an improved understanding of resonance and its neurovisceral correlates.

Heart-derived cues may contribute gaining an improved understanding of physiological resonance between subjects since it has been known for years that the brain and the heart exhibit permanent interactions, referred to as neurovisceral interactions here, that are critical beyond cardiovascular health, for behavioral, cognitive and emotion regulations [23,24,25,26,27]. The neurovisceral integration model first presented in 2000 [24] and then updated in 2009 [25] posits, based on the framework called the “central autonomic network” (CAN [23]), that a network of reciprocally connected brain regions is implicated in cardiac autonomic control. The characterization of the multiple circuits linking the heart and the brain by using autonomic indexes derived from heart rate variability (HRV) are thus believed to provide information about operating networks [23,25,26,27,28]. Both top-down (brain-to-heart) and bottom-up (heart-to-brain) aspects have been examined. HRV biofeedback training [29,30,31], a routine stimulating the baroreflex by slow breathing, shows that large oscillations in HRV, in any circumstances, influence neural networks, especially in brain regions regulating stress and emotions [32]. Concomitant top-down and bottom-up interactions maintain/improve functional connectivity in brain networks, spanning several temporal scales, which is likely reflected in some particular markers able to reflect multiplicative interactions at several scales.

Classically, HRV-derived metrics of autonomic functions are based on cardiac inter-beat time series, where successive inter-beat durations are obtained from the elapsed time between two successive R peaks in the electrocardiogram (ECG) signal. Parasympathetic (vagal) tone modulations are derived from the magnitude in the so-called high frequency fluctuations in the RR time series (±0.25 Hz), quite equivalently derived from power spectral density or the root mean square of successive differences (RMSSD) of HRV time series. Sympathetic arousals (despite potential contamination by vagal influence) are derived from the power of ±0.1 Hz fluctuations. Considering the interplay between cardiac vagal tone and top-down and bottom-up cognitive and emotional regulations, particular attention has been paid to parasympathetic indexes derived from the analysis of HRV time series [23,26,27,28,33]. Recently, there has been a growing appreciation that another class of HRV markers, derived from the fields of nonlinear dynamics may account for complexity in heart–brain interactions [34,35,36,37,38,39,40]. In the domain of complex systems theory, complexity in physiological systems is thought to reflect coordinated—in contrast with random or rigidly organized—interactions within a complex circuitry [37,38,41]. Complexity vanishes with ageing or impairments but is strengthened by cortical activities, as long as the system can cope with constraints. In the neural network, the presence of many structures interplaying at multiple hierarchical levels and at multiple time scales provides the system with complexity. The field of complex systems physiology has shown that behavioral complexity is reflected in HRV signal complexity. The latter can be captured with reasonable reliability by estimating the level of entropy in HRV dynamics [34]. The computations of these particular nonlinear HRV marker has been recommended in recent works to explore mood and cognition [40]. They were also shown to reflect how stress interacts with cognition [31,42,43].

The aim of the present study was to focus on nonlinear HRV dynamics, able to reflect multiple interactions across temporal scales that take place through both top-down and bottom-up communication between the brain and the heart, to gain a better understanding of physiological resonance associated with empathic stress. On the basis of our previous works [31,42,43], we hypothesized that the complexity marker of entropy would reflect an alteration in heart–brain interactions in targets experiencing stress as well as in observers, thus characterizing both first-hand stress and second-hand stress (empathic stress). To go further, we also hypothesized that this marker could highlight a physiological resonance phenomenon in observers (physiological linkage with the targets).

## 2. Materials and Methods

### 2.1. Participants

The study includes 100 participants randomly divided into 2 groups: the experimental (Exper) group (*n* = 82, 18.2 ± 0.9 years, 40 women, body mass index: 18.2 ± 0.9 kg/m^2^) and the control (Ctrl) group (*n* = 18, 18.4 ± 1.0 years, 10 women, body mass index: 18.4 ± 1 kg/m^2^). Randomization was performed using the formula *= alea()* in a spreadsheet, where participants were attributed a randomized number that determined the assigned group (Exper or Ctrl).

Participants were recruited among students in sport sciences. The exclusion criteria were the following: prior cardiovascular disease, severe inflammation, psychological disorders and medication intake affecting the cardiovascular system. Participants were requested not to consume alcohol and caffeinated drinks and to limit strenuous physical exercise the day before the experimentation.

All the participants (students) gave their written informed consent to participate in the study. Experiments were part of the students training program; they received credits for their participation. The whole procedure was approved by the institutional review board of the faculty who has entire responsibility on the training program.

### 2.2. Protocol

**Figure 1:**
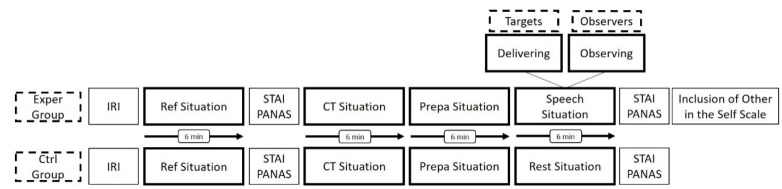
Flowchart of the experimental protocol.

The experimental protocol (Figure 1) took place between 9:00 am and 12:00 pm. Each experimental session simultaneously involved 3 participants belonging to the same group (either Exper or Ctrl). The participants were seated throughout the experimentation.

The protocol was designed as 4 successive situations each lasting 6 minutes: the reference situation (Ref), the cognitive task situation (CT), the speech preparation situation (Prepa) and the speech situation for Exper who were divided into target and observers (see below) or a rest situation for Ctrl.

The Ref situation aimed to evaluate a baseline psychophysiological status, participants were asked to relax, watching an emotionally neutral film. During the CT situation, participants performed a working memory task. This task consisted of a *n*-back paradigm (2-back and 3-back) designed in the same way as in the study of Schoofs et al. [44]. The Prepa and the speech situations referred to a modified Trier social stress test (TSST), a standardized laboratory stress task designed by Kirschbaum et al. [45]. In our protocol, all the participants (Exper and Ctrl) were instructed to imagine defending themselves to demonstrate their interest and their legitimacy in pursuing sport sciences studies. They had 6 min to prepare the speech (Prepa). At the end of this preparation period, a role was randomly assigned to each of the three Exper participants of a same experimental situation (speech): observer for two of them and target for the third. Observers were informed that they would not have to present the speech they prepared; they passively observed the third participant delivering his speech. During the speech situation, an audience of two people entered the room and the target had to deliver his speech in front of this audience and a video camera. Ctrl participants were invited to leave the room and stay calm and seated in a nearby space without talking.

### 2.3. Psychological Characteristics

Anxiety and affect were assessed in participant after Ref and speech/rest situations. To this end, participants filled out the Spielberger’s State-trait anxiety inventory (STAI) [46] and the positive and negative affect schedule (PANAS) [47].

Prior to the experimental protocol, participants completed the interpersonal reactivity index (IRI) [48], a multidimensional trait empathy index separating subscales for empathic concern, personal distress, perspective taking and fantasy. Although in the context of empathic stress we only focus on the empathy trait of the observers, all the participants filled out the IRI so that they cannot make assumptions about their future role in the following experimental situations.

After the experimental protocol (once observers and targets were identified), each observer indicated how close he felt with the target by choosing two circles whose intersection (or absence of intersection) coded their relationship closeness (i.e., their familiarity) following recommendations of the inclusion of other in the self scale [49].

### 2.4. Physiological Characteristics

Interbeat cardiac periods (RR) time series were recorded to evaluate HRV of the participants during the whole experimental setup. The time series were recorded with an accuracy of ±1 ms from a chest belt Polar H10 (Polar, Kempele, Finland) connected by Bluetooth to a smartphone application on iPod (Apple, Cupertino, CA, USA).

RR time series were transferred, *a posteriori*, to a computer. Artefacts (identified when the difference between two successive intervals was larger than 250 ms) were replaced by the average value of the nearby values. Then, markers of HRV in time-, frequency- and nonlinear domains were extracted using custom build algorithms in Matlab (Matlab 2019b, Matworks, Natick, MA, USA). To obtain a marker of signal complexity from nonlinear HRV dynamics, an enhanced method of multiscale entropy was used (see Section 2.4.2).

#### 2.4.1. Time-Domain and Frequency-Domain Cardiac Autonomic Markers

We computed the average RR duration (RR_mean_), the root mean square of successive differences between adjacent normal RR intervals (RMSSD) as well as the powers in some frequency bands after 4 Hz (regular) resampling of RR time series considering cubic spline interpolation. Based on power spectral density (PSD) obtained with discrete Fourier transform, low-frequency (LF) power (0.04 Hz to 0.15 Hz) and high-frequency (HF) power (0.15 Hz to 0.4 Hz) were computed [50]. The LF/HF ratio was calculated, as a marker of the sympathovagal balance [50].

#### 2.4.2. Complexity Marker of Heart Rate Variability Time Series

Multiscale entropy of RR interval time series (not resampled) was calculated from the refined composite multiscale entropy (RCMSE) method [51]. RCMSE is derived from the multiscale entropy (MSE) and the composite multiscale entropy (CMSE). Considering sample entropy over different scales, these methods allow to evaluate complexity in physiological time series [34]. The entropy value obtained at each scale depicts the average rate of information creation. The global level of complexity of a time series is computed by integrating the entropy values over a range of several scales. As explained by Wu et al. [51], RCMSE enhanced the precision of MSE (and CMSE) by decreasing the probability to obtain undefined entropy. To analyze short time series (as in the current work), the RCMSE method is highly advised [51].

Shortly, the RCMSE method includes the following steps (details are provided in [51]):

(1) The RR time series is coarse grained considering overlapping windows to represent the initial time series on several time scales τ. Overlapping windows lead to k coarse-grained series at each scale factor of τ.
(2) At each scale factor of τ, the matched vector pairs,$\;n_{k,\tau }^{m+1}$ and $n_{k,\tau \;}^{m},$ are counted for all (k) τ coarse-grained series, with m (m=2 in the present study) which corresponds to the sequence length chosen. This operation refers to the probability that vectors (sequences) of m samples that are similar, remain similar with the increase of the sequence length to m+1.
(3) At a scale factor of τ, RCMSE is computed as follows, with r, the tolerance value allowing to consider that vectors are matched. In the present work, r=0.15 of the standard deviation of the original time series:
  (1) $$RCMSE\left(x,\;\tau ,\;m,\;r\right)=-ln\left(\frac{\sum_{k=1}^{\tau }n_{k,\tau }^{m+1}}{\sum_{k=1}^{\tau }n_{k,\tau }^{m}}\right)\;\;\;\;$$

In our study, RCMSE was calculated over the range of scales 1 to 3, larger scales were excluded considering the likelihood of unreliable results [51,52]. The complexity marker (C_M_) was computed from the area under the curve of entropy vs. scale (using the trapezoidal rule) for the first three scales.

### 2.5. Statistical Analyses

All statistical analyses were conducted by use of R (R Core Team (2019), R: A language and environment for statistical computing, R Foundation for Statistical Computing, Vienna, Austria). Quantitative measurements are expressed as mean ± standard deviation. For clarity, data in the figures are depicted using standard error of the mean.

For anxiety scores, affect scores and cardiac markers, mixed model analyses of variance (ANOVA) with the role of participants (target vs. observer vs. Ctrl) as between factor and the situation (Ref vs. speech/rest for anxiety and affect and Ref vs. CT vs. Prepa vs. speech/rest for cardiac markers) as repeated factor were performed. Post-hoc Tukey correction was applied on pairwise comparisons within each ANOVA.

In order to further explore the main psychological and physiological explanatory variables potentially involved in the physiological stress response of the observers when they passively observed the targets delivering their (stressful) speech, a linear model (multiple linear regression) was established. To this end, the complexity marker change scores (ΔC_M_) of observers between Prepa and speech situations were entered as the dependent variable and psychological characteristics and physiological responses served as predictors. Because the multiple linear regression investigates only the main effects of predictors, assuming that the relationship between a given predictor (for example relationship closeness) and the dependent variable (ΔC_M_) is independent of the other predictor variables (anxiety, affect and complexity indexes), interaction effects were tested considering an additive model. This second model was computed by testing the possible interactions between predictor variables. Additionally, collinearity was evaluated to detect redundancy between predictor variables by computing the variance inflation factor (VIF) which measures how much the variance of a regression coefficient is inflated due to multicollinearity in the model. The small value of VIF (less than 1.2) in the present analysis indicated the absence of collinearity.

To examine the link between the trait empathy of observers and their physiological response, the ΔC_M_ of observers between Prepa and speech situations were correlated with the trait empathy scores per subscale (Pearson correlations).

## 3. Results

### 3.1. Psychological Characteristics

**Figure 2:**
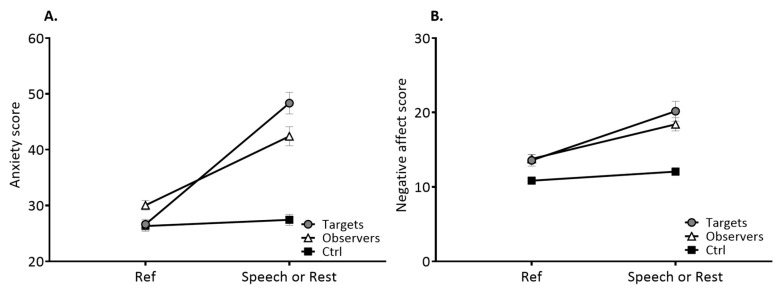
Anxiety (**A**) and negative affect (**B**) scores in reference (Ref) and speech/rest situations for the targets, observers and control (Ctrl) participants.

Considering the psychological responses, no differences were observed among both targets and observers while the latter were only passively observing the former during the speech situation (Figure 2). A significant role × situation effect was, respectively, found for anxiety (F(2, 97) = 26.62, *p* < 0.001) and negative affect (F(2, 97) = 3.76, *p* = 0.027). Targets and observers showed a significant increase in anxiety and negative affect (all *p* < 0.0001), whereas Ctrl participants did not (Figure 2).

No such changes were observed as regards positive affect, either between the groups, in a situation or an interaction manner.

The trait empathy main scores of the observers and the relationship closeness with targets are presented in Table 1. The empathy scores of observers were not different of those of targets and Ctrl participants (all *p* > 0.05, one-way ANOVA).

### 3.2. Physiological Characteristics

#### 3.2.1. Time-Domain and Frequency-Domain Cardiac Autonomic Markers

**Figure 3:**
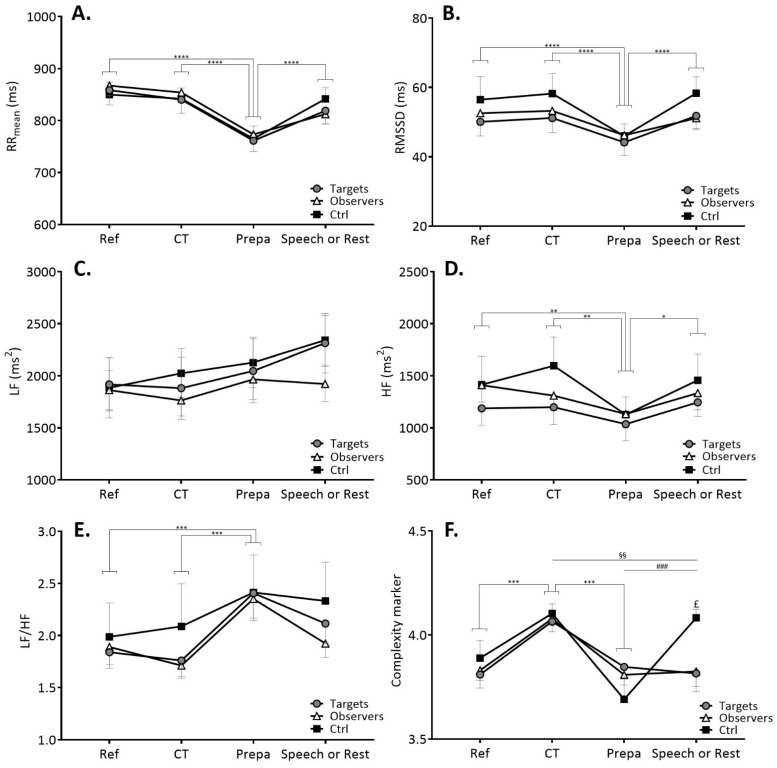
RR_mean_ (**A**), RMSSD (**B**), LF (**C**), HF (**D**), LF/HF (**E**) and complexity marker (**F**) mean values during reference (Ref), cognitive task (CT), speech preparation (Prepa) and speech/rest situations for targets, observers and control (Ctrl) participants. Significant differences correspond to the pairwise comparisons (Tukey post hoc) following the ANOVA results. **** *p* < 0.0001, *** *p* < 0.001, ** *p* < 0.01, * *p* < 0.05 for differences between experimental situations. $$ *p* < 0.01 for differences between experimental situations only for targets and observers. ### *p* < 0.001 for differences between situations only for Ctrl participants. £ *p* < 0.05 for differences between Ctrl participants and, respectively, targets and observers. RMSSD: root mean square of successive differences, LF: low-frequency power, HF: high-frequency power.

Classical cardiac autonomic markers obtained through time- and frequency-domain analyses typically failed to differentiate singular responses among targets, observers and Ctrl participants (no role or interaction effects, Figure 3). During Prepa, vagal markers (RMSSD, HF power) decreased while LF/HF increased (all *p* < 0.01). During the speech/rest situation vagal markers increased (all *p* < 0.05), while LF/HF remained elevated (Figure 3).

#### 3.2.2. Complexity in Heart Rate Variability Time Series

Where classical cardiac markers have failed to differentiate targets and/or observers from Ctrl participants, the complexity marker in the time series of HRV showed appealing group-specific profiles during the speech/rest situation. An overall role × situation effect was found (F(4.81, 223.67) = 3.21, *p* = 0.009). During the three first situations, a common profile for all groups was observed, that fluctuated over the situations. Multiscale entropy in HRV time series increased during CT and decreased during Prepa for all groups indistinctively (Figure 3F).

As a main result, entropy remained low in speech situation in both targets and observers whereas Ctrl participants restored a higher entropy (*p* < 0.001, Figure 3F). Here, the fact that observers response followed targets response where both differed from Ctrl, is critical for the analysis of physiological resonance between subjects.

### 3.3. Psychophysiological Keys in Observers-Targets Linkage

A multiple linear regression model was carried out for estimating the stress response of the observers between Prepa and speech situations based on multiple predictor variables. This way, the variation in cardiac entropy between Prepa and speech (∆C_M_ observers) was considered as the dependent variable. Predictor variables were the psychological and physiological variables measured in this study to avoid the arbitrary exclusion of a significant contributor. Regarding psychological variables, anxiety, affect and relationship closeness were entered into the model. Regarding physiological variables, based on our main hypothesis and in view of above results (Figure 3), only the complexity indexes were entered in the model.

The highest model significance (F(3, 46) = 11.84, R^2^ = 0.40, *p* < 0.0001) was observed with three predictors of the dependent variable (∆C_M_ observers). Indeed, among physiological and psychological markers, complexity indices (∆C_M_ targets between Prepa and speech, ∆C_M_ observers between CT and Prepa), as well as relationship closeness, exhibited significant linkage with ∆C_M_ of observers (Table 2). Hence, a significant amount of variance in the response of observers while they watched their counterparts delivering a speech was explained by their own response in Prepa situation, their measured relationship closeness with the target and, perhaps more importantly, the response of the observers was explained by the response of the targets, which is salient in our understanding of physiological resonance.

No association was found between observers trait empathy and their physiological stress response during the speech situation: no correlation was evidenced between the scores of empathic concern (r = 0.11, *p* = 0.44), personal distress (r = 0.07, *p* = 0.61), perspective taking (r = 0.15, *p* = 0.29) and fantasy (r = 0.23, *p* = 0.11) and the ΔC_M_ of observers between Prepa and speech situations.

## 4. Discussion

The aim of the present study was to demonstrate that empathic stress is associated to physiological resonance of heart rate variability complexity between targets and observers. In our conditions, when participants (targets) were directly exposed to the stress of delivering a speech, stress sharing was concurrently evidenced in the observers, in association with a blunted entropy of HRV dynamics. The present work represents the first evidence that impaired complexity in the output cardiac autonomic control is critical in stress resonance phenomenon.

Even if individuals perceive themselves as autonomous entities, their emotions and affective states are related to those of their peers, which facilitates social connection and coordination among human beings [53,54,55,56]. This emotional sharing is based on the human capacity of empathy that enables one to infer the state of another and to generate a similar state in the self [9,11,55,57,58]. Empathic stress, which refers to second-hand stress reactions in an observer, is a multifaced phenomenon that has raised psychosocial and physiological issues [1,2]. Empathy is, however, a broad conceptualization merging diverse phenomena going from perspective taking to sympathy, mimicry and emotional contagion [55]. As a possible consequence of an overly broad concept, self-reported trait empathy, evaluated through four facets, had no influence on the resonant empathic stress responses in the present study. In the present work, a more focused attention was paid to recent demonstrations that anxiety-induced changes in amygdala functioning deeply affect interconnectivity among large cortical–subcortical networks [59,60], which in turn may alter a coordinated organization in heart–brain interactions [42]. While first-hand stress has been associated with an altered complexity in the HRV output behavior of heart rate autonomic control, it is unknown to date if such a mechanism could operate with second-hand stress. Our results provide several evidences in agreement with this line of thought. First, we confirm the presence of emotional stress responses as a result of the speech task of the TSST. More importantly, anxiety and negative affect rose in both targets and observers suggesting emotional sharing and stress contagion between first-hand and second-hand stressed people (Figure 2). Second, time-domain and frequency-domain markers failed to differentiate targets and observers from control participants. This observation is in agreement with the lack of clear identification of stress contagion based on power in autonomic modulations of heart rate in previous works [1]. Rather, entropy appears as a reliable marker for understanding stress sharing. Strictly speaking, sample entropy evaluates irregularity in time series. When assessed over an adequate range of scales, sample entropy provides a reliable index of complexity in physiological time series [34]. When applied to the temporal structure of HRV fluctuations, entropy is supposed to reflect the complexity that emerges from coordinated interactions among neural networks that operate at several hierarchical and temporal scales to ultimately modulate heart rate in a complex manner [34]. In agreement with recent reports, index of complexity in cardiac dynamics increased during a cognitive task [42,43]. This is precisely what we observed here in all groups of participants. After that, entropy decreased during the phase of a speech preparation. This behavior fits well with the one reported previously when a cognitive task was followed by a cognitive task with added stressors [42]. It is thus suggested that during the phase wherein targets and observers naively prepared a speech, stress and anxiety as evidenced by psychological markers (Figure 2) were accompanied by an impaired coordination in heart–brain interactions reflected by the significant decrease in system complexity. Since observers were unable to restore a coordinated control while observing targets delivering a speech, we postulate that this behavior is a hallmark of physiological resonance. This result is unlikely to be fortuitous. Indeed, we observe that participants in the control group have a higher value of entropy when they leave the room and return to a non-stressful activity. It is therefore highly probable that the observers were contaminated by the stress of the targets, which confirms the physiological resonance between these participants demonstrated by the results of the multiple linear model.

As explained by Buchanan et al. [3] and Engert et al. [18], vicarious stress is suggested to arise through the projection of a target’s own stress response onto an observer, irrespective of the observer’s response. Conversely, concluding on the presence of stress resonance requires the level of the observer’s stress response to be a function of the level of the target’s stress response. Here we used the fit of a linear model of predictors of the observers stress to further explore individual fluctuations in entropy among targets and observers, thereby getting a finer appreciation of resonance. The linear model demonstrates that the entropy response in an observer depends on the entropy response in the target he observed, on his past own entropy and on the social closeness between the target and the observer. The significant role played by the entropy response of the target as a predictor in the linear model is a strong argument in favor of physiological resonance between the targets and the observers in our conditions. Empathic stress as an embodied autonomic process has already been suggested based on autonomic arousals [1,17,20], but not in terms involving the complex heart–brain interactions. In our conditions, neither sympathetic nor vagal modulations depicted the resonance. This might strengthen the significance of nonlinear markers that are able to account for complex dynamics, which has already been underlined in other domains, e.g., ageing [61], mood and cognition [39,40]. Here, we show that HRV entropy, as a nonlinear marker of complexity, may add significant value for the exploration of physiological resonance between subjects.

This study tried to further describe the role of neurophysiological resonance by emphasizing complexity in heart–brain interactions, but main outcomes might be strengthened. The extent to which complexity in heart rate autonomic control reflects complexity in brain networks connectivity cannot be demonstrated here; such a link could at best be inferred from recent evidence in the sensorimotor field [62]. Despite great potential in mapping brain functional connectivity to explore physiological resonance, such analyses were out of the scope of the present study and deserves further research.

## 5. Conclusions

The present study strengthens the relevance of using complexity markers to explore embodied autonomic control. A finer appraisal of complexity in heart–brain interactions is suggested a key factor for the exploration of short-term physiological resonance associated with emphatic stress.

## Figures and Tables

**Table 1:** Empathy main scores of observers and relationship closeness with targets.

**Trait Empathy (Interpersonal Reactivity Index)**	
Empathic concern	20.5 ± 4.2
Personal distress	11.7 ± 5.4
Perspective taking	16.1 ± 4.5
Fantasy	15.1 ± 5.8
Relationship closeness (inclusion of other in the self scale)	2.6 ± 1.8

**Table 2:** *ß* coefficients with corresponding *p*-values for significant predictor variables obtained from the multiple linear regression model (dependent variable: ∆C_M_ of observers).

	*β*	*p* Value
ΔC_M_ targets between Prepa and speech situations	0.42	0.0089
ΔC_M_ observers between CT and Prepa situations	−0.85	0.0003
Relationship closeness of observers with targets	−3.96	0.0003

ΔC_M_: complexity marker change scores, Prepa: speech preparation, CT: cognitive task.
